# Psychology’s Status as a Science: Peculiarities and Intrinsic Challenges. Moving Beyond its Current Deadlock Towards Conceptual Integration

**DOI:** 10.1007/s12124-020-09545-0

**Published:** 2020-06-17

**Authors:** Jana Uher

**Affiliations:** School of Human Sciences, University of Greenwich, Old Royal Naval College, Park Row, London, SE10 9LS UK

**Keywords:** Experience, Terminology, Soft Science, Nomothetic, Construct, Integrative framework

## Abstract

Psychology holds an exceptional position among the sciences. Yet even after 140 years as an independent discipline, psychology is still struggling with its most basic foundations. Its key phenomena, mind and behaviour, are poorly defined (and their definition instead often delegated to neuroscience or philosophy) while specific terms and constructs proliferate. A unified theoretical framework has not been developed and its categorisation as a ‘soft science’ ascribes to psychology a lower level of scientificity. The article traces these problems to the peculiarities of psychology’s study phenomena, their interrelations with and centrality to everyday knowledge and language (which may explain the proliferation and unclarity of terms and concepts), as well as to their complex relations with other study phenomena. It shows that adequate explorations of such diverse kinds of phenomena and their interrelations with the most elusive of all—immediate experience—inherently require a plurality of epistemologies, paradigms, theories, methodologies and methods that complement those developed for the natural sciences. Their systematic integration within just one discipline, made necessary by these phenomena’s joint emergence in the single individual as the basic unit of analysis, makes psychology in fact the hardest science of all. But Galtonian nomothetic methodology has turned much of today’s psychology into a science of populations rather than individuals, showing that blind adherence to natural-science principles has not advanced but impeded the development of psychology as a science. Finally, the article introduces paradigmatic frameworks that can provide solid foundations for conceptual integration and new developments.

## Psychology’s Status as a Discipline

Psychology holds an exceptional position among the sciences—not least because it explores the very means by which any science is made, for it is humans who perceive, conceive, define, investigate, analyse and interpret the phenomena of the world. Scientists have managed to explore distant galaxies, quantum particles and the evolution of life over 4 billion years—phenomena inaccessible to the naked eye or long deceased. Yet, psychology is still struggling with its most basic foundations. The phenomena of our personal experience, directly accessible to everyone in each waking moment of life, remain challenging objects of research. Moreover, psychical phenomena are essential for all sciences (e.g., thinking). But why are we struggling to scientifically explore the means needed to first make any science? Given the successes in other fields, is this not a contradiction in itself?

This article outlines three key problems of psychology (poor definitions of study phenomena, lack of unified theoretical frameworks, and an allegedly lower level of scientificity) that are frequently discussed and at the centre of Zagaria, Andò and Zennaro’s (2020) review. These problems are then traced to peculiarities of psychology’s study phenomena and the conceptual and methodological challenges they entail. Finally, the article introduces paradigmatic frameworks that can provide solid foundations for conceptual integration and new developments.

### Lack of Proper Terms and Definitions of Study Phenomena

Introductory text books are supposed to present the corner stones of a science’s established knowledge base. In psychology, however, textbooks present definitions of its key phenomena—mind (psyche) and behaviour—that are discordant, ambiguous, overlapping, circular and context-dependent, thus inconclusive (Zagaria et al. 2020). Tellingly, many popular text books define ‘mind’ exclusively as ‘brain activity’, thus turning psychology’s central object of research into one of neuroscience. What then is psychology as opposed to neuroscience? Some even regard the definition of mind as unimportant and leave it to philosophers, thus categorising it as a philosophical phenomenon and shifting it again out of psychology’s own realm. At the same time, mainstream psychologists often proudly distance themselves from philosophers (Alexandrova & Haybron, 2016), explicitly referring to the vital distinction between science and philosophy. Behaviour, as well, is commonly reduced to ill-defined ‘activities’, ‘actions’ and ‘doings’ and, confusingly, often even equated with mind (psyche), such as in concepts of ‘inner and outer behaviours’ (Uher 2016b). All this leaves one wonder what psychology is actually about.

As if to compensate the unsatisfactory definitional and conceptual status of its key phenomena in general, psychology is plagued with a chaotic proliferation of terms and constructs for specific phenomena of mind and behaviour (Zagaria et al. 2020). This entails that different terms can denote the same concept (jangle-fallacies; Kelley 1927) and the same terms different concepts (jingle-fallacies; Thorndike 1903). Even more basically, many psychologists struggle to explain what their most frequent study phenomena—constructs—actually are (Slaney and Garcia 2015). These deficiencies and inconsistencies involve a deeply fragmented theoretical landscape.

### Lack of Conceptual Integration Into Overarching Frameworks

Like no other science, psychology embraces an enormous diversity of established epistemologies, paradigms, theories, methodologies and methods. Is that a result of the discipline’s unparalleled complexity and the therefore necessary scientific pluralism (Fahrenberg 2013) or rather an outcome of mistaking this pluralism for the unrestrained proliferation of perspectives (Zagaria et al. 2020)?

The lack of a unified theory in psychology is widely lamented. Many ‘integrative theories’ were proposed as overarching frameworks, yet without considering contradictory presuppositions underlying different theories. Such integrative systems merely provide important overviews of the essential plurality of research perspectives and methodologies needed in the field (Fahrenberg 2013; Uher 2015b). Zagaria and colleagues (2020) suggested evolutionary psychology could provide the much-needed paradigmatic framework. This field, however, is among psychology’s youngest sub-disciplines and its most speculative ones because (unlike biological phenomena) psychical, behavioural and social phenomena leave no fossilised traces in themselves. Their possible ancestral forms can only be reconstructed indirectly from archaeological findings and investigations of today’s humans, making evolutionary explorations prone to speculations and biases (e.g., gender bias in interpretations of archaeological findings; Ginge 1996). Cross-species comparative psychology offers important correctives through empirical studies of today’s species with different cognitive, behavioural, social and ecological systems and different degrees of phylogenetic relatedness to humans. This enables comparisons and hypothesis testing not possible when studying only humans but still faces limitations given human ancestors’ unavailability for direct study (Uher 2020a).

But most importantly, evolutionary psychology does not provide consistent terms and concepts either; its key constructs ‘psychological adaptations’ and ‘evolved psychological mechanisms’ are as vague, ambiguous and ill-defined as ‘mind’ and ‘behaviour’. Moreover, the strong research heuristic formulated in Tinbergen’s four questions on the causation, function, development and evolution of behaviour is not an achievement of evolutionary psychology but originates from theoretical biology, thus again from outside of psychology.

### Psychology—a ‘Soft Science’ in Pre-scientific Stage?

The pronounced inconsistencies in psychology’s terminological, conceptual and theoretical landscape have been likened to the pre-scientific stage of emerging sciences (Zagaria et al. 2020). Psychology was therefore declared a ‘soft science’ that can never achieve the status of the ‘hard sciences’ (e.g., physics, chemistry). This categorisation implies the belief that some sciences have only minor capacities to accumulate secured knowledge and lower abilities to reach theoretical and methodological consensus (Fanelli and Glänzel 2013; Simonton 2015). In particular, soft sciences would have only limited abilities to apply ‘the scientific method’, the general set of principles involving systematic observation, experimentation and measurement as well as deduction and testing of hypotheses that guide scientific practice (Gauch 2015). The idea of the presumed lack of methodological rigor and exactitude of ‘soft sciences’ goes back to Kant (1798/2000) and is fuelled by recurrent crises of replication, generalisation, validity, and other criteria considered essential for all sciences.

But classifying sciences into ‘hard’ and ‘soft’, implying some would be more scientific than others, is ill-conceived and misses the point why there are different sciences at all. Crucially, the possibilities for implementing particular research practices are not a matter of scientific discipline or their ascribed level of scientificity but solely depend on the particular study phenomena and their properties (Uher 2019). For study phenomena that are highly context-dependent and continuously changing in themselves, such as those of mind, behaviour and society, old knowledge cannot have continuing relevance as this is the case for (e.g., non-living) phenomena and properties that are comparably invariant in themselves. Instead, accurate and valid investigations require that concepts, theories and methods must be continuously adapted as well (Uher 2020b).

The classification of sciences by the degree to which they can implement ‘the scientific method’ as developed for the natural sciences is a reflection of the method-centrism that has taken hold of psychology over the last century, when the craft of statistical analysis became psychologists’ dominant activity (Lamiell 2019; Valsiner 2012). The development of ever more sophisticated tools for statistical analysis as well as of rating scales enabling the efficient generation of allegedly quantitative data for millions of individuals misled psychologists to adapt their study phenomena and research questions to their methods, rather than vice versa (Omi 2012; Toomela and Valsiner 2010; Uher 2013). But methods are just a means to an end. Sciences must be phenomenon-centred and problem-centred, and they must develop epistemologies, theories, methodologies and methods that are suited to explore these phenomena and the research problems in their field.

## Psychology’s Study Phenomena and Intrinsic Challenges

Psychology’s exceptional position among the sciences and its key problems can be traced to its study phenomena’s peculiarities and the conceptual and methodological challenges they entail.

### Experience: Elementary to All Empirical Sciences

Experience is elementary to all empirical sciences, which are experience-based by definition (from *Greek* empeiria meaning experience). The founder of psychology, Wilhelm Wundt, already highlighted that every concrete experience has always two aspects, the objective content given and individuals’ subjective apprehension of it—thus, the objects of experience in themselves and the subjects experiencing them. This entails two fundamental ways in which experience is treated in the sciences (Wundt 1896a).

Natural sciences explore the objective contents mediated by experience that can be obtained by subtracting from the concrete experience the subjective aspects always contained in it. Hence, natural scientists consider the objects of experience in their properties as conceived independently of the subjects experiencing them, using the perspective of *mediate experience* (mittelbare Erfahrung; Wundt 1896a). Therefore, natural scientists develop theories, approaches and technologies that help minimise the involvement of human perceptual and conceptual abilities in research processes and filter out their effects on research outcomes. This approach is facilitated by the peculiarities of natural-science study phenomena (of the non-living world, in particular), in which general laws, immutable relationships and natural constants can be identified that remain invariant across time and space and that can be measured and mathematically formalised (Uher 2020b).

Psychologists, in turn, explore the experiencing subjects and their understanding and interpretation of their experiential contents and how this mediates their concrete experience of ‘reality’. This involves the perspective of *immediate experience* (unmittelbare Erfahrung), with immediate indicating absence of other phenomena mediating their perception (Wundt 1896a). Immediate experience comprises connected processes, whereby every process has an objective content but is, at the same time, also a subjective process. Inner experience, Wundt highlighted, is not a special part of experience but rather constitutes the entirety of all immediate experience; thus, inner and outer experience do not constitute separate channels of information as often assumed (Uher 2016a). That is, psychology deals with the entire experience in its immediate subjective reality. The inherent relation to the perceiving and experiencing subject—*subject reference*—is therefore a fundamental category in psychology. Subjects are feeling and thinking beings capable of intentional action who pursue purposes and values. This entails agency, volition, value orientation and teleology. As a consequence, Wundt highlighted, research on these phenomena can determine only law-like generalisations that allow for exceptions and singularities (Fahrenberg 2019). Given this, it is meaningless to use theories-to-laws ratios as indicators of scientificity (e.g., in Simonton 2015; Zagaria et al. 2020).

### Constructs: Concepts in Science AND Everyday Psychology

The processual and transient nature of immediate experience (and many behaviours) imposes further challenges because, of processual entities, only a part exists at any moment (Whitehead 1929). Experiential phenomena can therefore be conceived only through generalisation and abstraction from their occurrences over time, leading to concepts, beliefs and knowledge *about them*, which are psychical phenomena in themselves as well but different from those they are about (reflected in the terms experien*cing* versus experien*ce*; Erleben versus Erfahrung; Uher 2015b, 2016a). Abstract concepts, because they are theoretically constructed, are called *constructs* (Kelly 1963). All humans implicitly develop constructs (through abduction, see below) to describe and explain regularities they observe in themselves and their world. They use constructs to anticipate the unknown future and to choose among alterative actions and responses (Kelly 1963; Valsiner 2012).

Constructs about experiencing, experience and behaviour form important parts of our everyday knowledge and language. This entails intricacies because psychologists cannot simply put this everyday psychology aside for doing their science, even more so as they are studying the phenomena that are at the centre of everyday knowledge and largely accessible only through (everyday) language. Therefore, psychologists cannot invent scientific terms and concepts that are completely unrelated to those of everyday psychology as natural scientists can do (Uher 2015b). But this also entails that, to first delineate their study phenomena, psychologists need not elaborate scientific definitions because everyday psychology already provides some terms, implicit concepts and understanding—even if these are ambiguous, discordant, circular, overlapping, context-dependent and biased. This may explain the proliferation of terms and concepts and the lack of clear definitions of key phenomena in scientific psychology.

Constructs and language-based methods entail further challenges. The construal of constructs allowed scientists to turn abstract ideas into entities, thereby making them conceptually accessible to empirical study. But this *entification* misguides psychologists to overlook their constructed nature (Slaney and Garcia 2015) by ascribing to constructs an ontological status (e.g., ‘traits’ as psychophysical mechanisms; Uher 2013). Because explorations of many psychological study phenomena are intimately bound to language, psychologists must differentiate their study phenomena from the terms, concepts and methods used to explore them, as indicated by the terms psych*ical* versus psych*ological* (from Greek -λογία, -logia for body of knowledge)—differentiations not commonly made in the English-language publications dominating in contemporary psychology (Lewin 1936; Uher 2016a).

### Psychology’s Exceptional Position Among the Sciences and Philosophy

The concepts of mediate and immediate experience illuminate psychology’s special interrelations with the other sciences and philosophy. Wundt conceived the *natural sciences* (Naturwissenschaften; e.g., physics, physiology) as auxiliary to psychology and psychology, in turn, as supplementary to the natural sciences “in the sense that only together they are able to exhaust the empirical knowledge accessible to us“ (Fahrenberg 2019; Wundt 1896b, p. 102). By exploring the universal forms of immediate experience and the regularities of their connections, psychology is also the foundation of the *intellectual sciences* (Geisteswissenschaften, commonly (mis)translated as humanities; e.g., philology, linguistics, law), which explore the actions and effects emerging from humans’ immediate experiences (Fahrenberg 2019). Psychology also provides foundations for the *cultural and social sciences* (Kultur- und Sozialwissenschaften; e.g., sociology; anthropology), which explore the products and processes emerging from social and societal interactions among experiencing subjects who are thinking and intentional agents pursuing values, aims and purposes. Moreover, because psychology considers the subjective and the objective as the two fundamental conditions underlying theoretical reflection and practical action and seeks to determine their interrelations, Wundt regarded psychology also a preparatory empirical science for *philosophy* (especially epistemology and ethics; Fahrenberg 2019).

Psychology’s exceptional position at the intersection with diverse sciences and with philosophy is reflected in the extremely heterogeneous study phenomena explored in its diverse sub-disciplines, covering all areas of human life. Some examples are individuals’ sensations and perceptions of physical phenomena (e.g., psychophysics, environmental psychology, engineering psychology), biological and pathological phenomena associated with experience and behaviour (e.g., biopsychology, neuropsychology, clinical psychology), individuals’ experience and behaviour in relation to others and in society (e.g., social psychology, personality psychology, cultural psychology, psycholinguistics, economic psychology), as well as in different periods and domains of life (e.g., developmental psychology, educational psychology, occupational psychology). No other science explores such a diversity of study phenomena. Their exploration requires a plurality of epistemologies, methodologies and methods, which include experimental and technology-based investigations (e.g., neuro-imaging, electromyography, life-logging, video-analyses), interpretive and social-science investigations (e.g., of texts, narratives, multi-media) as well as investigations involving self-report and self-observation (e.g., interviews, questionnaires, guided introquestion).

All this shows that psychology cannot be a unitary science. Adequate explorations of so many different kinds of phenomena and their interrelations with the most elusive of all—immediate experience—inherently require a plurality of epistemologies, paradigms, theories, methodologies and methods that complement those developed for the natural sciences, which are needed as well. Their systematic integration within just one discipline, made necessary by these phenomena’s joint emergence in the single individual as the basic unit of analysis, makes psychology in fact the hardest science of all.

### Idiographic and Nomothetic Strategies of Knowledge Generation

Immediate experience, given its subjective, processual, context-dependent, and thus ever-changing nature, is always unique and unprecedented. Exploring such *particulars* inherently requires *idiographic* strategies, in which local phenomena of single cases are modelled in their dynamic contexts to create generalised knowledge from them through abduction. In abduction, scientists infer from observations of surprising facts backwards to a possible theory that, if it were true, could explain the facts observed (Peirce 1901; CP 7.218). Abduction leads to the creation of new general knowledge, in which theory and data are circularly connected in an open-ended cycle, allowing to further generalise, extend and differentiate the new knowledge created. By generalising from what was once and at another time as well, idiographic approaches form the basis of *nomothetic* approaches, which are aimed at identifying generalities *common to all* particulars of a class and at deriving theories or laws to account for these generalities. This Wundtian approach to nomothetic research, because it is *case-by-case based*, allows to create generalised knowledge about psychical processes and functioning, thus building a bridge between the individual and theory development (Lamiell 2003; Robinson 2011; Salvatore and Valsiner 2010).

But beliefs in the superiority of natural-science principles misled many psychologists to interpret nomothetic strategies solely in terms of the Galtonian methodology, in which many cases are aggregated and statistically analysed on the *sample-level*. This limits research to *group-level* hypothesis testing and theory development to inductive generalisation, which are uninformative about single cases and cannot reveal what is, indeed, common to all (Lamiell 2003; Robinson 2011). This entails numerous fallacies, such as the widespread belief between-individual structures would be identical to and even reflect within-individual structures (Molenaar 2004; Uher 2015d). Galtonian nomothetic methodology has turned much of today’s psychology into a science exploring populations rather than individuals. That is, blind adherence to natural-science principles has not advanced but, instead, substantially impeded the development of psychology as a science.

## Moving Psychology Beyond its Current Conceptual Deadlock

Wundt’s opening of psychology’s first laboratory marked its official start as an independent science. Its dynamic developments over the last 140 years testify to psychology’s importance but also to the peculiarities of its study phenomena and the intricate challenges that these entail for scientific explorations. Yet, given its history, it seems unlikely that psychology can finally pull itself out of the swamps of conceptual vagueness and theoretical inconsistencies using just its own concepts and theories, in a feat similar to that of the legendary Baron Münchhausen. Psychology can, however, capitalise on its exceptional constellation of intersections with other sciences and philosophy that arises from its unique focus on the individual. Although challenging, this constitutes a rich source for perspective-taking and stimulation of new developments that can meaningfully complement and expand its own genuine achievements as shown in the paradigm outlined now.

### The Transdisciplinary Philosophy-of-Science Paradigm for Research on Individuals (TPS-Paradigm)

The *Transdisciplinary Philosophy-of-Science Paradigm for Research on Individuals* (*TPS-Paradigm*^2^) is targeted toward making explicit and scrutinising the most basic assumptions that different disciplines make about research on individuals to help scientists critically reflect on; discuss and refine their theories and practices; and to derive ideas for new developments (therefore *philosophy-of–science*). It comprises a system of interrelated philosophical, metatheoretical and methodological frameworks that coherently build upon each other (therefore *paradigm*). In these frameworks, concepts from various lines of thought, both historical and more recent, and from different disciplines (e.g., psychology, life sciences, social sciences, physical sciences, metrology, philosophy of science) that are relevant for exploring research objects in (relation to) individuals were systematically integrated, refined and complemented by novel ones, thereby creating unitary frameworks that transcend disciplinary boundaries (therefore *transdisciplinary*; Uher 2015a, b, 2018c).

#### The Philosophical Framework: Presuppositions About Research on Individuals

The philosophical framework specifies three sets of presuppositions that are made in the TPS-Paradigm about the nature and properties of individuals and the phenomena studied in (relations to) them as well as about the notions by which knowledge about them can be gained.

1. *All science is done by humans* and therefore inextricably entwined with and limited by human’s perceptual and conceptual abilities. This entails risks for particular fallacies of the human mind (e.g., oversimplifying complexity, Royce 1891; reifying linguistic abstractions, Whitehead 1929). Scientists researching individuals face particular challenges because they are individuals themselves, thus inseparable from their research objects. This entails risks for anthropocentric, ethnocentric and egocentric biases influencing metatheories and methodologies (Uher 2015b). Concepts from social, cultural and theoretical psychology, sociology, and other fields (e.g., Gergen 2001; Valsiner 1998; Weber 1949) were used to open up meta-perspectives on research processes and help scientists reflect on their own presuppositions, ideologies and language that may (unintentionally) influence their research.
2. *Individuals are complex living organisms*, which can be conceived as open (dissipative) and nested systems. On each hierarchical level, they function as organised wholes from which new properties emerge not predictable from their constituents and that can feed back to the constituents from which they emerge, causing complex patterns of upward and downward causation. With increasing levels of organisation, ever more complex systems emerge that are less rule-bound, highly adaptive and historically unique. Therefore, dissecting systems into elements cannot reveal the processes governing their functioning and development as a whole; assumptions on universal determinism and reductionism must be rejected. Relevant concepts from thermodynamics, physics of life, philosophy, theoretical biology, medicine, psychology, sociology and other fields (e.g., Capra 1997; Hartmann 1964; Koffka 1935; Morin 2008; Prigogine and Stengers 1997; Varela et al. 1974; von Bertalanffy 1937) about dialectics, complexity and nonlinear dynamic systems were used to elaborate their relevance for research on individuals.
3. *The concept of complementarity* is applied to highlight that, by using different methods, ostensibly incompatible information can be obtained about properties of the same object of research that are nevertheless all equally essential for an exhaustive account of it and that may therefore be regarded as complementary to one another. Applications of this concept, originating from physics (wave-particle dilemma in research on the nature of light; Bohr 1937; Heisenberg 1927), to the body-mind problem emphasise the necessity for a methodical dualism to account for observations of two categorically different realities that require different frames of reference, approaches and methods (Brody and Oppenheim 1969; Fahrenberg 1979, 2013; Walach 2013). Complementarity was applied to specify the peculiarities of psychical phenomena and to derive methodological concepts (Uher, 2016a). It was also applied to develop solutions for the nomothetic-idiographic controversy in ‘personality’ research (Uher 2015d).

These presuppositions underlie the metatheoretical and the methodological framework.

#### Metatheoretical Framework

The metatheoretical framework formalises a phenomenon’s accessibility to human perception under everyday conditions using three metatheoretical properties: internality-externality, temporal extension, and spatiality conceived complementarily as physical (spatial) and “non-physical” (without spatial properties). The particular constellations of their forms in given phenomena were used to metatheoretically define and differentiate from one another various kinds of phenomena studied in (relation to) individuals: morphology, physiology, behaviour, psyche, semiotic representations (e.g., language), artificial outer-appearance modifications (e.g., clothing) and contexts (e.g., situations; Uher 2015b).

These metatheoretical concepts allowed to integrate and further develop established concepts from various fields to elaborate the peculiarities of the phenomena of the psyche^3^ and their functional connections with other phenomena (e.g., one-sided psyche-externality gap; Uher 2013), to trace their ontogenetic development and to explore the fundamental imperceptibility of others’ psychical phenomena and its role in the development of agency, language, instructed learning, culture, social institutions and societies in human evolution (Uher 2015a). The metatheoretical definition of behaviour^4^ enabled clear differentiations from psyche and physiology, and clarified when the content-level of language in itself constitutes behaviour, revealing how language extends humans’ behavioural possibilities far beyond all non-language behaviours (Uher 2016b). The metatheoretical definition of ‘personality’ as individual-specificity in all kinds of phenomena studied in individuals (see above) highlighted the unique constellation of probabilistic, differential and temporal patterns that merge together in this concept, the challenges this entails and the central role of language in the formation of ‘personality’ concepts. This also enabled novel approaches for conceptual integrations of the heterogeneous landscape of paradigms and theories in ‘personality’ research (Uher 2015b, c, d, 2018b). The semiotic representations concept emphasised the composite nature of language, comprising psychical and physical phenomena, thus both internal and external phenomena. Failure to consider the triadic relations among meaning, signifier and referent inherent to any sign system as well as their inseparability from the individuals using them was shown to underly various conceptual fallacies, especially regarding data generation and measurement (Uher 2018a, 2019).

#### Methodological Framework

The metatheoretical framework is systematically linked to the methodological framework featuring three main areas.

1. *General concepts of phenomenon-methodology matching*. The three metatheoretical properties were used to derive implications for research methodology, leading to new concepts that help to identify fallacies and mismatches (e.g., nunc-ipsum methods for transient phenomena, intro*questive* versus extro*questive* methods to remedy methodological problems in previous concepts of introspection; Uher 2016a, 2019).
2. *Methodological concepts* for comparing individuals within and across situations, groups and species were developed (Uher 2015e). *Approaches for taxonomising individual differences* in various kinds of phenomena in human populations and other species were systematised on the basis of their underlying rationales. Various novel approaches, especially behavioural ones, were developed to systematically test and complement the widely-used lexical models derived from everyday language (Uher 2015b, c, d, 2018b, c).
3. *Theories and practices of data generation and measurement* from psychology, social sciences and metrology, the science of measurement and foundational to the physical sciences, were scrutinised and compared. These transdisciplinary analyses identified two basic methodological principles of measurement underlying metrological concepts that are also applicable to psychological and social-science research (data generation traceability, numerical traceability; Uher 2020b). Further analyses explored the involvement of human abilities in data generation across the empirical sciences (Uher 2019) and raters’ interpretation and use of standardised assessment scales (Uher 2018a).

Empirical demonstrations of these developments and analyses in various empirical studies involving humans of different sociolinguistic backgrounds as well as several nonhuman primate species (e.g., Uher 2015e, 2018a; Uher et al. 2013a, b; Uher and Visalberghi 2016) show the feasibility of this line of research. Grounded in established concepts from various disciplines, it offers many possibilities for fruitful cross-scientific collaborations waiting to be explored in order to advance the fascinating science of individuals.

## Footnotes

2
http://researchonindividuals.org.

3 The psyche is defined as the “entirety of the phenomena of the immediate experiential reality both conscious and non-conscious of living organisms” (Uher 2015c, p. 431, derived from Wundt 1896a).

4 Behaviours are defined as the “external changes or activities of living organisms that are functionally mediated by other external phenomena in the present moment” (Uher 2016b, p. 490).
